# Scintillation in (C_6_H_5_CH_2_NH_3_)_2_SnBr_4_: green-emitting lead-free perovskite halide materials†

**DOI:** 10.1039/d1ra01123e

**Published:** 2021-06-10

**Authors:** Lina Jaya Diguna, Silmi Kaffah, Muhammad Haris Mahyuddin, Arramel, Francesco Maddalena, Suriani Abu Bakar, Mimin Aminah, Djulia Onggo, Marcin Eugeniusz Witkowski, Michal Makowski, Winicjusz Drozdowski, Muhammad Danang Birowosuto

**Affiliations:** School of Applied Science, Technology, Engineering, and Mathematics, Universitas Prasetiya Mulya Kavling Edutown I.1, Jl. BSD Raya Utama, BSD City Tangerang 15339 Indonesia lina.diguna@prasetiyamulya.ac.id; Research Group of Advanced Functional Materials, Research Center for Nanoscience and Nanotechnology, Institut Teknologi Bandung Jl. Ganesha 10 Bandung 40132 Indonesia haris@tf.itb.ac.id; Department of Physics, National University of Singapore 2 Science Drive 3 Singapore 117551 Singapore; CINTRA UMI CNRS/NTU/THALES 3288 Research Techno Plaza, 50 Nanyang Drive, Border X Block, Level 6 Singapore 637553 Singapore mbirowosuto@ntu.edu.sg; Nanotechnology Research Centre, Faculty of Science and Mathematics, Universiti Pendidikan Sultan Idris 35900 Tanjung Malim Perak Malaysia; Inorganic and Physical Chemistry Research Group, Faculty of Mathematics and Natural Sciences, Institut Teknologi Bandung Jl. Ganesha 10 Bandung 40132 Indonesia; Institute of Physics, Faculty of Physics, Astronomy, and Informatics, Nicolaus Copernicus University in Torun ul. Grudziadzka 5 Torun 87-100 Poland

## Abstract

We report the optical and scintillation properties of (C_6_H_5_CH_2_NH_3_)_2_SnBr_4_ with excellent absorption length at 20 keV of 0.016 cm, measured bandgap of 2.51 eV, and photoluminescence lifetime of 1.05 μs. The light yield obtained with the ^241^Am source is 3600 ± 600 photons per MeV, which is much smaller than the maximum attainable light yield obtained from the bandgap. Temperature dependent radioluminescence measurements confirm the presence of thermal quenching at room temperature with the activation energy and the ratio between the attempt and the radiative transition rates of 61 meV and 129, respectively. Although thermal quenching affects light yield at room temperature, this green light-emitting perovskite opens an avenue for new lead-free scintillating materials.

## Abstract 2

A new tin perovskite material with green emission is investigated as a new scintillator for imaging readout. Temperature dependent measurements were studied to understand the mechanism and to improve the future lead-free scintillator.

## Graphical abstract

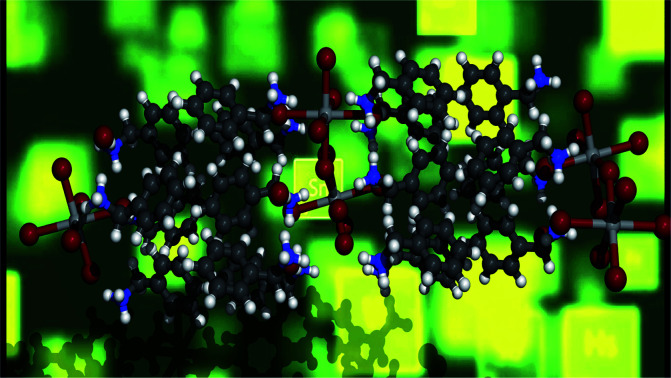

## Introduction

Lead halide perovskite materials gain lots of interest as a new generation of scintillating materials.^1–4^ The reported compounds are commonly hybrid organic–inorganic perovskite (HOIP) lead halide crystals^4–7^ or all-inorganic perovskite (AIP) lead halide quantum dots^2,8^ and nanosheets.^9^ For HOIP lead halide crystals, the main application is low-energy radiation scintillators, *e.g.* X-ray, nevertheless recently they were shown to be effective for gamma-ray and neutron spectroscopy with good energy resolution.^6,7^ Although larger effective atomic number and mass density should favor AIP lead halide quantum dots and nanosheets for stopping high-energy radiation applications, their radiation absorption lengths are in fact much smaller than those in bulks. The short actual lengths are due to the presences of the ligands and/or adhesives making the packing fractions much lower than those in bulks.^2,8,9^ Also, small Stokes shift generates some problems with the reabsorption.^10^ For HOIP lead halide crystals, the bromide crystals are more stable and having larger light yields than iodide ones.^7^ However, the blue emission of bromide crystals is less attractive due to the lower quantum efficiencies for typical photodiodes used for X-ray imaging display panels.^11^ Finally, both HOIP and AIP lead halide materials share the same disadvantage as the lead cation (Pb) is toxic and can cause severe health hazard.

The possible cation replacement for lead is manganese (Mn),^12^ copper (Cu),^13,14^ tin (Sn),^15^ antimony (Sb),^16^ bismuth (Bi),^17^ and europium (Eu).^18^ With the cation replacement, beside decreasing the toxicity, we also can tune the emission wavelength from blue to green. However, all of them, except Bi, have effective atomic numbers and atomic masses lower than Pb reducing the radiation absorption. Despite that scintillation efficiency for most lead free perovskite materials has been estimated to be lower than for Pb compounds,^19,20^ it has to be verified by means of pulse height spectra and radioluminescence measurements. In this work, we report optical and scintillation properties of green-light-emitting (C_6_H_5_CH_2_NH_3_)_2_SnBr_4_ crystals as a new potential candidate of lead free scintillator. The photoluminescence (PL) peak is at 498.14 nm while time-resolved PL (TRPL) decay curve exhibits the lifetime of 1.05 μs. For scintillation, we compare its properties to the previous sibling, (CH_3_(CH_2_)_7_NH_3_)_2_SnBr_4_ (ref. 15) as it seems quite bright for X-ray imaging. Since the organic chains in (C_6_H_5_CH_2_NH_3_)_2_SnBr_4_ are much shorter than those in (CH_3_(CH_2_)_7_NH_3_)_2_SnBr_4_, we obtain that the respective absorption length at 20 keV of 0.016 cm is slightly shorter than that of 0.017 cm. The bandgap of (C_6_H_5_CH_2_NH_3_)_2_SnBr_4_ is found to be 2.51 and 2.12 eV for experimental and theoretical values, respectively. For the empirical light yield calculation considering unity photoluminescence quantum yields (PLQY) and transfer efficiencies,^4^ the maximal attainable light yield is inversely proportional to the bandgap and therefore, the value for (C_6_H_5_CH_2_NH_3_)_2_SnBr_4_ is expected to be between 160 000 and 190 000 photons per MeV. However, the light yield at room temperature (RT) is relatively low (3600 ± 600 photons per MeV) and we associate this low yield due to low PLQY and thermal quenching of the exciton as it is a common observation in HOIP crystals.^4^ For parameters in quenching, we compare both activation energy and the ratio between thermal quenching and radiative transition rates in other HOIP crystals.^12^ This should lead to more in-depth understanding of scintillation processes in lead-free HOIP.

## Experimental methods

### Synthesis of (C_6_H_5_CH_2_NH_3_)_2_SnBr_4_

Hydrobromic acid (HBr, ACS reagent, 48%), hypophosphorous acid (H_3_PO_2_, 50 wt% in H_2_O), benzylamine (C_6_H_5_CH_2_NH_2_, ReagentPlus, 99%) and tin(ii) bromide (SnBr_2_) used for synthesis, were purchased from Sigma-Aldrich. The synthesis method of (C_6_H_5_CH_2_NH_3_)_2_SnBr_4_ was adopted and modified from the reference reported by Cao *et al.*^15^ The compound was prepared from aqueous solution of 2 ml C_6_H_5_CH_2_NH_2_ and 3 ml of 48% HBr in 5 ml distilled water mixed with a solution of 0.5 g SnBr_2_ in 7 ml of 48% HBr and 20 ml of H_3_PO_2_. The solution mixture was stirred vigorously and heated to 60 °C for three hours to achieve a homogeneous solution. The crystals were grown after the mixture was left over few days at room temperature. The crystals were filtered from the solution mixture, and dried in aeration. For microcrystals, we kept the crystals in the solution mixture (initial precursors) and put them in the ultrasonic bath operating at 80 kHz for 15 minutes. All samples were kept in the dry box and only to be exposed with air directly prior and after measurements.

### X-ray diffraction

Powder X-ray diffraction (XRD) data were recorded on a Bruker D8 Advance AXS diffractometer with a graphite-monochromatized Cu Kα radiation (*λ* = 1.54178 Å). The data acquisition was employed at RT under Bragg–Brentano geometry with the speed of scanning of 10 s per step and step size of 0.02.

### Scanning electron and atomic force microscopes

Microstructure observation was done by using a scanning electron microscope (SEM, LEO 1550 Gemini, Carl Zeiss AG, Oberkochen, Germany) with an accelerating voltage of 5 kV and a commercial atomic force microscope (AFM, BRUKER Dimension FastScan). A standard cantilever with spring constant of 40 N m^−1^ and tip curvature < 10 nm was used as the probe.

### X-ray photoelectron spectroscopy

X-ray photoelectron spectroscopy (XPS) experiments were carried out by using X-ray source of magnesium Kα with typical excitation energy output of 1254 eV. The impinged spot size on the sample is about 1 mm in diameter.

### Photoluminescence, time-resolved photoluminescence, and absorption

PL measurements were performed at room temperature (RT) using free-space excitation and collection through a visible-near-infrared microscope objective (Nikon 20×, Nikon Corporation, Tokyo, Japan, NA = 0.40). The sample was excited *via* a 30 kHz picosecond pulsed diode laser (Master Oscillator Fibre Amplifier, Picoquant, Picoquant GmbH, Berlin, Germany, excitation wavelength at 355 nm, pulse width 50 ps, and power of 10 μW). The PL measurement was based on epifluorescence method. PL spectra were detected using AvaSpec-HERO spectrometer (Avantes BV, Apeldoorn, The Netherlands). The emission was then selected by a band filter at 500 ± 25 nm and detected by a single-photon avalanche photodiode (APD) connected to a time-correlated single-photon counting acquisition module (Edinburgh Instruments, TCC900, Edinburgh Instruments Ltd., Livingstone, United Kingdom). Absorption spectra of perovskite crystals were obtained using ultraviolet-visible (UV-vis) spectrometer (Shimadzu, Model UV-2450). PLQY measurements were carried out by placing the sample inside a Labsphere integrating sphere coupled to a Newton 920 Charge Coupled Device (Andor) through an optical fibre for photon counting. Cobolt 320 nm continuous-wave diode laser was used as an excitation source.

### Radioluminescence, thermoluminescence, glow curves, and imaging

For all those measurements, we used one integrated setup. It consists of an Inel XRG3500 X-ray generator Cu-anode tube, 45 kV/10 mA, an Acton Research Corporation SpectraPro-500i monochromator, a Hamamatsu R928 photomultiplier tube (PMT), and an APD Cryogenic Inc. closed-cycle helium cooler. First, we measured the radioluminescence (RL) at different temperatures between 10 and 350 K starting from the highest to lowest temperatures in order to avoid the possible contributions from thermal release of charge carriers to the emission yield. After RL measurements, we recorded low temperature afterglow and thermoluminescence (TL) glow curves. Prior TL measurements, the crystals were exposed to X-rays for 10 minutes at 10 K and the monochromator was set to the zeroth order. The glow curves were recorded at temperatures between 10 and 350 K with a heating rate of about 0.14 K s^−1^. For imaging, X-ray source was PHYWE XR 4.0 expert unit (Mo source, 17.5 keV) operated at 35 kV voltage and 1 mA current while the camera was a commercial color camera with 1 s exposure time. The card with the chip was just put in front of the scintillator while its back side was put close to the aperture of the X-ray tube where the uncollimated X-ray came out. They were placed as close as possible to reduce light scattering.

### Pulse height measurements

For the pulse height measurements, the crystal was mounted on the window of a Hamamatsu R9880U-20 PMT, with a thin layer of silicone grease to provide optical coupling. The PMT was operated at a voltage of −750 V for these measurements. The PMT anode signal was input directly to a Caen DT5720D digital pulse processing unit.

### Density functional theory calculations

The orthorhombic unit cell of (CH_3_(CH_2_)_7_NH_3_)_2_SnBr_4_ retrieved from Cao *et al.*^15^ and the triclinic unit cell of (C_6_H_5_(CH_2_)_2_NH_3_)_2_SnBr_4_ retrieved from Xu *et al.*^21^ were used for the (CH_3_(CH_2_)_7_NH_3_)_2_SnBr_4_ and (C_6_H_5_CH_2_NH_3_)_2_SnBr_4_ calculations, respectively. The Kohn–Sham formulation^22,23^ as implemented in the Vienna *Ab initio* Simulation Package (VASP)^24,25^ was used for the calculations. The Projector Augmented Wave (PAW) method^26,27^ was employed to describe the interaction between ion cores and electrons. The electron exchange–correlation was treated by the generalized gradient approximation (GGA) based on the Perdew–Burke–Ernzerhof (PBE) functional.^28^ A rotationally invariant GGA+U approach introduced by Dudarev *et al.*^29^ was used with an effective Hubbard parameter *U*_eff_ being 9.0 eV for the Sn p orbital. The plane wave basis sets with a cut-off energy of 500 eV were employed for all calculations. The Brillouin zone was sampled with 3 × 3 × 1, 3 × 3 × 3, 3 × 3 × 3 *k*-point grids for (CH_3_(CH_2_)_7_NH_3_)_2_SnBr_4_, (C_6_H_5_(CH_2_)_2_NH_3_)_2_SnBr_4_, and (C_6_H_5_CH_2_NH_3_)_2_SnBr_4_, respectively, according to the Monkhorst–Pack scheme.^30^ DFT-D3 method was adopted to account for the dispersion correction.^31^ The conjugate gradient method was employed for cell optimizations, and the calculations were considered to converge when the maximum forces on each atom were less than 0.01 eV/1 Å. During calculations, all atoms were allowed to be fully relaxed.

## Results and discussion

**Fig. 1:**
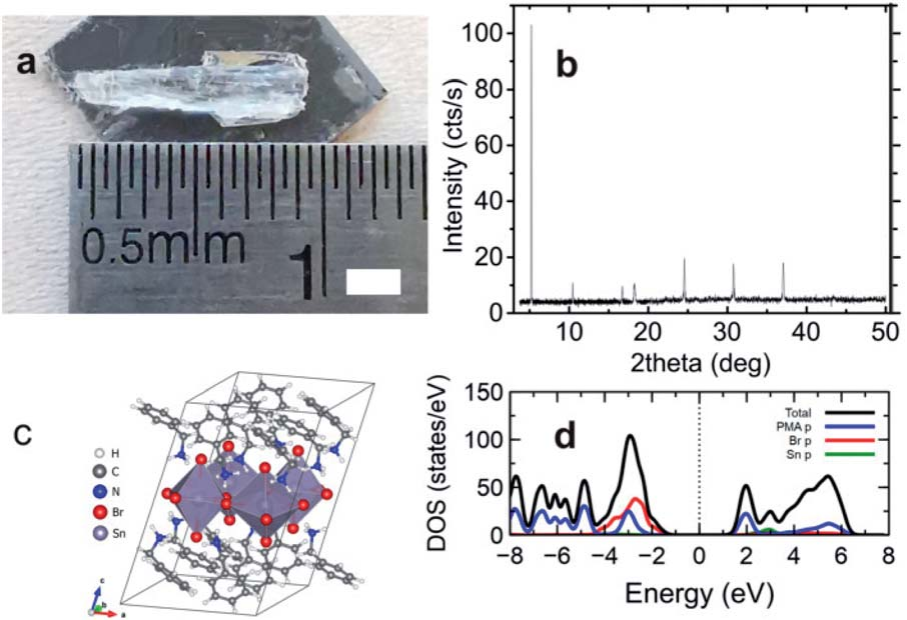
Appearance and structure of (C_6_H_5_CH_2_NH_3_)_2_SnBr_4_. (a) A photograph of a large crystal with a scale bar of 2 mm. (b) X-ray diffraction pattern. (c) Crystal structure representation. (d) Total (black) and projected (color) density of states (DOS). Blue, green, and red lines represent (C_6_H_5_CH_2_NH_3_) s and p, Sn p, and Br p orbitals, respectively.

Fig. 1 exhibits the details on the appearance and the structure of (C_6_H_5_CH_2_NH_3_)_2_SnBr_4_ crystals. The appearance of the single crystal and the X-ray diffraction (XRD) pattern are shown in Fig. 1a and b, respectively. The XRD lines were well fitted with the presumptuous model built from the (C_6_H_5_(CH_2_)_2_NH_3_)_2_SnBr_4_ structure^21,32^ with the removal of one [CH_2_] moiety in its ligand, see Fig. S1 and Table S1 of ESI.† Here we note that the diffraction patterns of (C_6_H_5_CH_2_NH_3_)_2_SnBr_4_ are 5.52°, 12.68°, 18.54°, 24.84°, 27.08°, 31.05°, and 37.34° which could be assigned to the (001), (002), (003), (004), (005), (006), and (007) lattice planes, respectively, comparable to the previous reported of 2D layered metal halide perovskite.^33–36^ For the complete calculated lattice and geometrical parameters comparing to those of (CH_3_(CH_2_)_7_NH_3_)_2_SnBr_4_ and (C_6_H_5_(CH_2_)_2_NH_3_)_2_SnBr_4_, we enlist them in Table S2 of ESI.† At RT, the crystal structure (shown in Fig. 1c) has triclinic phase with a space group of *P*1. This phase still belongs to the general class of A_2_SnX_4_ (X = I, Br, Cl and A = organic cation) HOIP crystals and consists of the stack of 〈100〉-oriented perovskite inorganic layers, forming a two-dimensional (2D) Sn-X octahedra network in alternation with the organic sheets of C_6_H_5_CH_2_NH_3_^+^ cations. The structure has a deficit of [CH_2_] chain in the cation of (C_6_H_5_(CH_2_)_2_NH_3_)_2_SnBr_4_ (ref. 32) and this will result a slightly short absorption length of 0.016 cm at 20 keV, see Fig. S2a of ESI.† For the estimation of theoretical light yield, we calculate the optical bandgap *E*_g_ (ref. 1 and 4) from the calculated density of states (DOS) using density functional theory (DFT) in Fig. 1d. We obtain calculated *E*^cal^_g_ of 2.12 eV for (C_6_H_5_CH_2_NH_3_)_2_SnBr_4_ while those for (C_6_H_5_(CH_2_)_2_NH_3_)_2_SnBr_4_ (ref. 21) and (CH_3_(CH_2_)_7_NH_3_)_2_SnBr_4_ (ref. 15) are 2.40 eV to 2.47 eV, respectively, see Fig. S2b and c of ESI.† Since (C_6_H_5_CH_2_NH_3_)_2_SnBr_4_ has the smallest *E*_g_, it has the highest estimated maximum light yield of approximately 190 000 photons per MeV.^1,4^

**Fig. 2:**
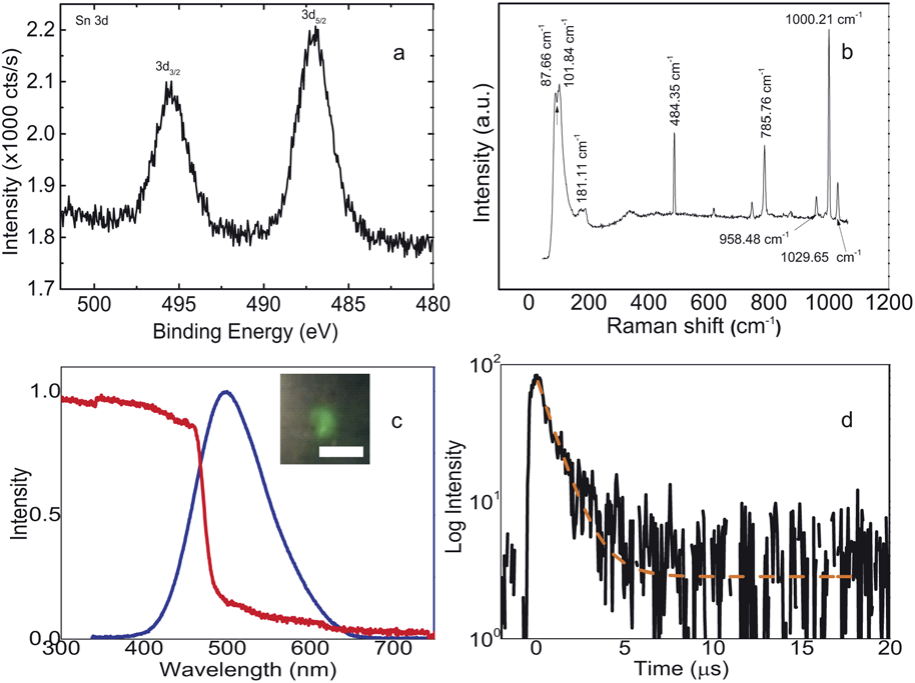
Surface characteristics and optical properties of (C_6_H_5_CH_2_NH_3_)_2_SnBr_4_. (a) X-ray photoelectron spectroscopy signals from Sn 3d levels and their corresponding binding energy. (b) Raman profile with the identification of each elements shown by the corresponding shifts. (c) Absorption and photoluminescence (PL) (excited at 266 nm) spectra recorded at room temperature (RT) shown in red and blue lines, respectively. The inset shows the PL image with a scale bar of 5 μm. (d) Time-resolved PL decay curve excited at 266 nm monitoring 500 nm emission.

Furthermore, the investigation on the quality of the crystals also involves the surface characteristics and elements inside (C_6_H_5_CH_2_NH_3_)_2_SnBr_4_ as shown in Fig. 2. The X-ray photoelectron spectroscopy (XPS) result in Fig. 2a shows peaks at 495.45 eV and 487.15 eV corresponding to the respective binding energies of tin (Sn) 3d_3/2_ and 3d_5/2_ core levels. Those binding energies are only 0.5 eV lower than those of (C_6_H_5_(CH_2_)_2_NH_3_)_2_SnBr_4_ reported by Xu *et al.*^21^ The chemical states of Sn^2+^ of (C_6_H_5_CH_2_NH_3_)_2_SnBr_4_ remains the same throughout the prolonged observation. We monitored the progression changes from the Sn^2+^ into Sn^4+^ oxidation states after three-month duration kept at ambient conditions. The rest of XPS signals can be found in Fig. S3 of ESI.† Raman spectrum shown in Fig. 2b exhibits several peaks at low frequencies less than 200 cm^−1^, one peak at 484.35 cm^−1^ due to SiO_2_ substrate, and three other peaks at frequencies around 1000 cm^−1^. The first two low frequency peaks at 87.66 and 101.84 cm^−1^ are attributed to two external modes of C_6_H_5_CH_2_NH_3_^+^.^37^ The peak centered at 181.11 cm^−1^ is attributed to the internal asymmetric stretching mode of Sn–Br vibration. Finally, the peaks above 500 cm^−1^ only arise from the internal modes of the organic cations.^37,38^

Absorption and PL spectra of (C_6_H_5_CH_2_NH_3_)_2_SnBr_4_ are shown in Fig. 2c. The peak for the PL is located at 498.14 nm. This is at ∼100 nm shorter wavelength than 596 nm peak of (CH_3_(CH_2_)_7_NH_3_)_2_SnBr_4_ (ref. 15) and ∼30 nm longer wavelength than 470 nm peak of (C_6_H_5_(CH_2_)_2_NH_3_)_2_SnBr_4_.^21^ For five days, the peak and the width of the PL almost remain constant while the intensity decreases by 23%, see Fig. S4 of ESI.† The large difference in the PL shift with (CH_3_(CH_2_)_7_NH_3_)_2_SnBr_4_ can be related with the completely different structure while the difference with (C_6_H_5_(CH_2_)_2_NH_3_)_2_SnBr_4_ is consistent with previously observed in Mn-based HOIP.^12^ For the latter, the longer emission wavelength is related to the lower exciton binding energy in (C_6_H_5_CH_2_NH_3_)_2_SnBr_4_ in comparison with the energy in (C_6_H_5_(CH_2_)_2_NH_3_)_2_SnBr_4_ due to the absence of one [CH_2_] chain in the cation. From the absorption spectra, we can derive the optical bandgap as it is shown in the Tauc plot of Fig. S5 from ESI.† The obtained bandgap is 2.51 eV and it is 0.23 eV larger than the bandgap calculated by DFT in Fig. 1b. This discrepancy with the same order was also observed in (C_6_H_5_(CH_2_)_2_NH_3_)_2_SnBr_4_ which the experimental and theoretical values are 2.67 eV and 2.40 eV, respectively.^21,32^ The experimental bandgap in (C_6_H_5_CH_2_NH_3_)_2_SnBr_4_ is still lower than those in (C_6_H_5_(CH_2_)_2_NH_3_)_2_SnBr_4_ and (CH_3_(CH_2_)_7_NH_3_)_2_SnBr_4_, which is expected from the shortest organic chain in (C_6_H_5_CH_2_NH_3_)_2_SnBr_4_. For Stokes shift, the values for (C_6_H_5_CH_2_NH_3_)_2_SnBr_4_ and (C_6_H_5_(CH_2_)_2_NH_3_)_2_SnBr_4_ are similar of 0.02 eV and 0.03 eV, respectively. Time resolved PL (TRPL) decay curve in Fig. 2d shows a lifetime of 1.05 μs, which is faster than those of 3.34 μs to 2.70 μs in (CH_3_(CH_2_)_7_NH_3_)_2_SnBr_4_ (ref. 15) and (C_6_H_5_(CH_2_)_2_NH_3_)_2_SnBr_4_,^21^ respectively.

**Fig. 3:**
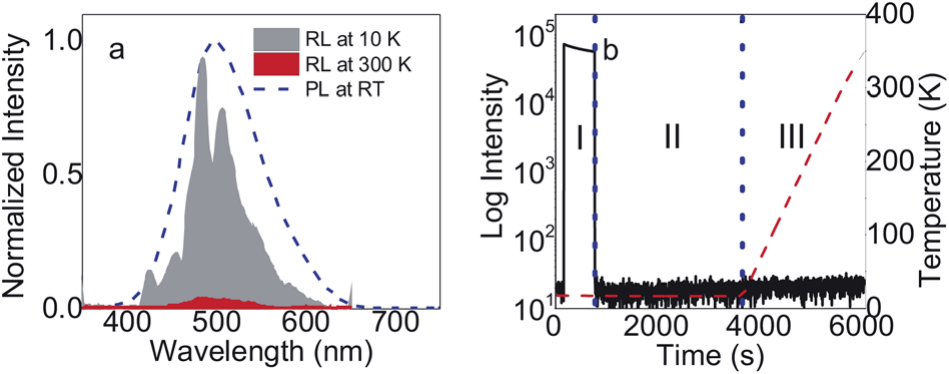
Radio- (RL) and thermoluminescence (TL) of (C_6_H_5_CH_2_NH_3_)_2_SnBr_4_. (a) RL spectra at 10 K and 300 K in comparison with PL at RT. (b) Steady state RL (I), after glow (II), and temperature-dependent TL (III) regions in trap-characteristic glow curve separated by blue dotted lines. After glow parts were recorded after 10 minutes of X-ray irradiation at 10 K while TL parts were measured with heating rate of 0.14 K s^−1^ indicated by red-dashed lines.

Radioluminescence (RL) and thermoluminescence (TL) properties are shown in Fig. 3. For RL in Fig. 3a, the spectra have the same center and width as that in PL. The spectra at 10 K exhibit more complex structure with peaks at 424.85, 454.29, 473.56, 484.31, 505.81, 543.18, 579.98 nm, respectively. Those peaks can be assigned with different types of excitons and possibility of more phases at low temperatures. For TL in Fig. 3b, very short afterglow and no traps were observed in (C_6_H_5_CH_2_NH_3_)_2_SnBr_4_. The absence of traps is related with very short chain of (C_6_H_5_CH_2_NH_3_)_2_SnBr_4_ in comparison with other 2D HOIP crystals.^12^ Thus, for scintillation mechanism, most excitons are free and therefore this material has the fastest lifetimes among other Sn HOIP crystals.^15,21^ These are the advantages of (C_6_H_5_CH_2_NH_3_)_2_SnBr_4_ for scintillator applications.

**Fig. 4:**
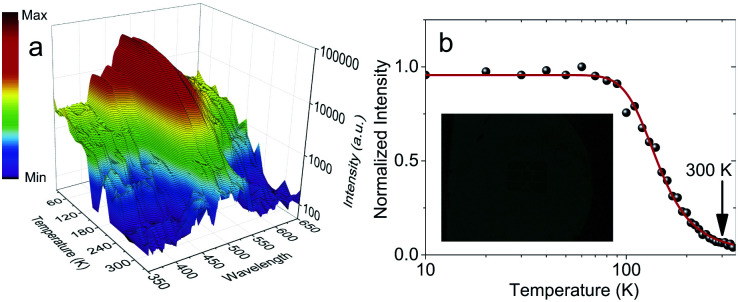
Temperature dependent RL of (C_6_H_5_CH_2_NH_3_)_2_SnBr_4_. (a) RL spectra mapping at different temperatures from 10 K to 350 K. (b) Normalized total spectrum integrated RL intensities at various temperatures from 10 K to 350 K. The red line exhibits the Arrhenius fit while the inset shows the X-ray image of a card chip inside an envelope using the film made from microcrystals.

Temperature dependent RL spectra mapping at different temperatures from 10 K to 350 K are shown in Fig. 4a. At low temperature, many RL peaks clearly appear, showing a good correlation with those observed in Fig. 3a. With the increase of temperature, those peaks vanish, overlap and are hardly to distinguish above 100 K. Those peaks are also hardly to separate in temperature-dependent PL spectra, see Fig. S6 of ESI.† We highlight that the peaks at 424.85 nm and 454.29 nm indicate the association of donor bound excitons, and they become less contributed at 200 K and above.^39^ The characteristics from the thermal quenching are presented as Arrhenius plot in Fig. 4b. Here, the activation energy derived by fitting the integrated RL intensities (*I*) with Arrhenius equation can be summarized as following:1
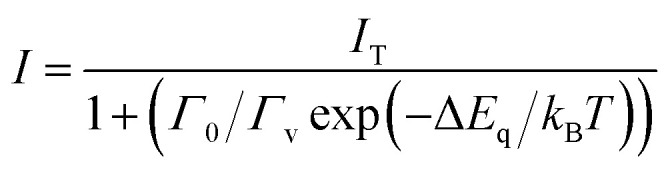
where *I*_T_, Δ*E*_q_, *Γ*_0_/*Γ*_v_, and *k*_B_ are the intensity at low temperature of around 10 K, the thermal quenching activation energy, the ratio between thermal quenching rate at *T* = ∞ (attempt rate) and the radiative transition rate, and Boltzmann constant, respectively. Eqn (1) fits well with the temperature dependence of RL intensities of (C_6_H_5_CH_2_NH_3_)_2_SnBr_4_ crystal. As it is only related to one mechanism without any assumption of the phase transitions, the smooth behavior of intensities reflects that the crystals have no phase transition. For (C_6_H_5_CH_2_NH_3_)_2_SnBr_4_, Δ*E*_q_ and *Γ*_0_/*Γ*_v_ are 61 meV and 129, respectively. *Γ*_0_/*Γ*_v_ of (C_6_H_5_CH_2_NH_3_)_2_SnBr_4_ is much smaller than those for (C_6_H_5_(CH_2_)_2_NH_3_)_2_MnCl_4_ (ref. 12) showing that the quenching of (C_6_H_5_CH_2_NH_3_)_2_SnBr_4_ happens at higher temperatures. We also note that the thermal quenching maybe responsible for 1.05 μs fast lifetime of (C_6_H_5_CH_2_NH_3_)_2_SnBr_4_ as it was previously observed in lanthanide trihalides.^40^

It was shown at Fig. 4b that at room temperature there is only 6.39% left of 10 K scintillation light. The low scintillation light yield is expected as the PLQY at RT is 4.83%, see Fig. S7 of ESI.† Such PLQY is larger than 0.10% of (C_6_H_5_CH_2_NH_3_)_2_SnBr_4_.^21^ Moreover, PLQY value is consistent with both temperature-dependent PL and TRPL measurements in Fig. S6 of ESI.† Both measurements show that PL intensity and lifetime at RT are 20.83% and 12.02% of those at 100 K, respectively. Consequently, the maximal attainable light yield with current PLQY becomes 9100 photons per MeV.^1^ Pulse height measurement with ^241^Am at RT in Fig. S8 of ESI† exhibits a light yield of 3600 ± 600 photons per MeV which corresponds to 56 000 ± 9000 photons per MeV at 10 K. Both experimental (3600 ± 600 photons per MeV) and new maximal attainable light yield (9100 photons per MeV) are still in agreement as the transfer efficiency still needs to be estimated.^1^ The light yield at 10 K is still consistent with the previous measurements on HOIP crystals at 10 K.^4^ With this light yield, we still can perform X-ray imaging as shown in the inset of Fig. 4b. For this measurement, we used 155 ± 23 μm-thick film composed by microcrystals in lognormal-distribution sizes with an average diagonal diameter of 0.45 μm, see Fig. S9 and S10 of ESI.† However, the sensitivity of this imaging is apparently still below that of (C_6_H_5_(CH_2_)_2_NH_3_)_2_SnBr_4_.^15^

## Conclusion

In this work, we reported optical and X-ray scintillation properties of (C_6_H_5_CH_2_NH_3_)_2_SnBr_4_ crystals prepared by solution process. The crystal shows bandgap of 2.51 eV, green emission with PL peak at 498.14 nm and fast PL lifetime at 500 nm of 1.05 μs. For scintillation, the observed short absorption length at 20 keV of 0.016 cm unexpectedly results the low light yield at RT (3600 ± 600 photons per MeV). From temperature dependent RL observation, the thermal quenching activation energy and the ratio between thermal quenching and radiative transition rates are 61 meV and 129, respectively. Compared to other HOIP crystals, the small ratio indicates the occurrence of thermal quenching at high temperature thus affecting the light yield. Although we still could perform X-ray imaging, the quenching effect should be minimized to increase the sensitivity. Another method to increase the light output is through the fabrication of the nanostructures as it was already demonstrated in perovskite materials.^41^ With these findings, (C_6_H_5_CH_2_NH_3_)_2_SnBr_4_ may be considered to open possibilities for new candidates of green-emitting lead-free perovskite scintillator.

## Author contributions

Sample preparation, L. J. D., D. O., and M. A.; optical characterization, PLQY, XRD, atomic force microscope (AFM), scanning electron microscope (SEM) and X-ray imaging, F. M., S. A. B., and M. D. B.; XPS and Raman characterization, A.; DFT characterization, M. H. M.; RL and TL characterization, M. E. W., M. M., and W. D.; data analysis and writing, L. J. D. and S. K.; supervision, W. D. and M. D. B. All authors have read and agreed to the published version of the manuscript.

## Conflicts of interest

There are no conflicts to declare.

## Supplementary Material

RA-011-D1RA01123E-s001
