# Supplementary material for: Scintillation in (C6H5CH2NH3)2SnBr4: green-emitting lead-free perovskite halide materials
Source: RSC Adv. 2021 Jun 10;11(34):20635–40. doi: 10.1039/d1ra01123e (PMC9034015; doi:10.1039/d1ra01123e)
Supplement: RA-011-D1RA01123E-s001 [file RA-011-D1RA01123E-s001.pdf]

Electronic Supplementary Material (ESI) for RSC Advances.
This journal is © The Royal Society of Chemistry 2021

Supplementary Information

Scintillation in $(C_6H_5CH_2NH_3)_2SnBr_4$: Green-Emitting Lead-Free Perovskite Halide Materials

L. J. Diguna,[1, a)] S. Kaffah,[1] M. H. Mahyuddin,[2, b)] Arramel,[3] F. Maddalena,[4] S. A. Bakar,[5] M. Aminah,[6] D. Onggo,[6] M. E. Witkowski,[7] M. Makowski,[7] W. Drozdowski,[7] and M. D. Birowosuto[4, c)]

[1)] *School of Applied Science, Technology, Engineering, and Mathematics, Universitas Prasetiya Mulya, Kavling Edutown I.1, Jl. BSD Raya Utama, BSD City, Tangerang 15339, Indonesia.*

[2)] *Research Group of Advanced Functional Materials and Research Center for Nanoscience and Nanotechnology, Institut Teknologi Bandung, Jl. Ganesha 10, Bandung 40132, Indonesia.*

[3)] *Department of Physics, National University of Singapore, 2 Science Drive 3 Singapore 117551, Singapore.*

[4)] *CINTRA UMI CNRS/NTU/THALES 3288, Research Techno Plaza, 50 Nanyang Drive, Border X Block, Level 6, Singapore 637553, Singapore.*

[5)] *Nanotechnology Research Centre, Faculty of Science and Mathematics, Universiti Pendidikan Sultan Idris 35900 Tanjung Malim, Perak, Malaysia.*

[6)] *Inorganic and Physical Chemistry Research Group, Faculty of Mathematics and Natural Sciences, Institut Teknologi Bandung, Jl. Ganesha 10, Bandung 40132, Indonesia.*

[7)] *Institute of Physics, Faculty of Physics, Astronomy, and Informatics, Nicolaus Copernicus University in Torun, ul. Grudziadzka 5, Torun 87-100, Poland.*

(Dated: 27 May 2021)

In this supplementary information, we explained the structure analysis, absorption length calculation, density functional theory calculation, photoluminescence and time-resolved luminescence analysis, quantum yield, radioluminescence and thermoluminescence analysis, X-ray diffraction analysis, X-ray photoelectron spectroscopy analysis, optical bandgap determination, pulse height measurements, scanning electron microscopy, and atomic force microscopy.

[a] Electronic mail: lina.diguna@prasetiyamulya.ac.id
[b] Electronic mail: haris@tf.itb.ac.id
[c] Electronic mail: mbirowosuto@ntu.edu.sg

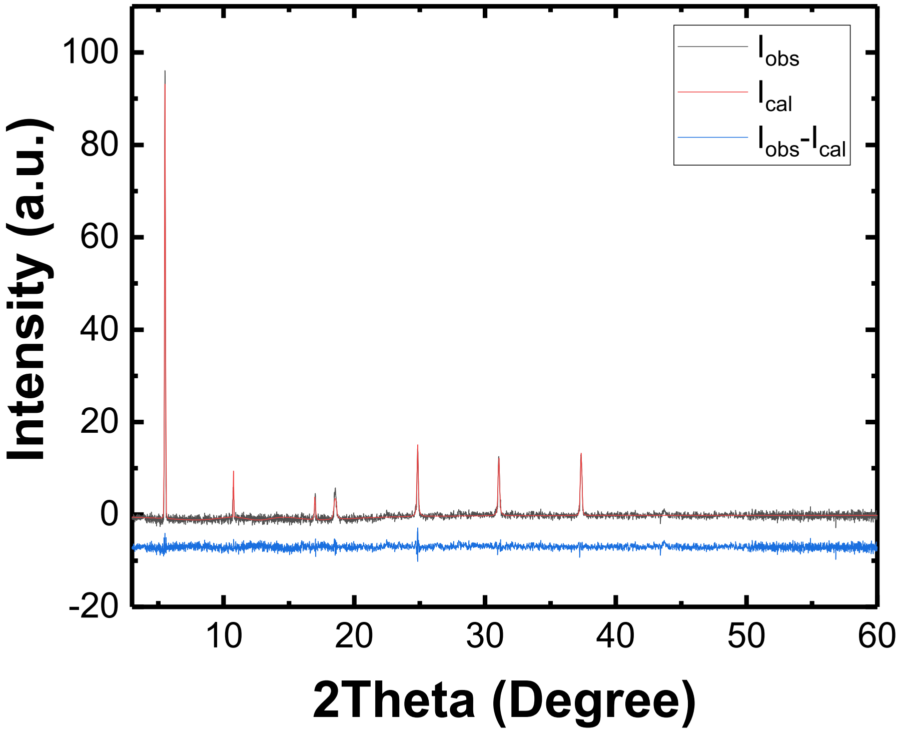

Fig. S1. Experimental I_{obs} and simulated I_{cal} XRD patterns of $(C_6H_5CH_2NH_3)_2SnBr_4$. The difference between the simulated and the experimental peaks is denoted $I_{obs} - I_{cal}$.

We used Origin 2019 software (https://www.originlab.com/2019) for the fitting procedure. The refinement analysis was performed based on the nonlinear curve fitting by using the model of Pseudo-Voigt function. The iteration process was done based on the Levenberg-Marquardt algorithm, where the formula can be found as follows:

$$y = y_0 + A\left(\frac{2\mu}{\pi}\frac{w}{4(x-x_c)^2+w^2}\right) + (1-\mu)\left(\frac{w\sqrt{4\ln 2}}{\sqrt{\pi}}\right)\exp\left(\frac{-4\ln 2}{w^2}(x-x_c)^2\right).$$

As a result, we collected the following fitting output in Table S1. The fit iteration in Figure S2 was performed 55 times, with total iteration in session of 60. The number of points is 5701 while we set the degree of freedom to 56.72. From the fit, we obtain the goodness χ^2 of 0.81, coefficient of determination R^2 of 0.98, and adjusted R^2 of 0.98. For $\chi^2 \lesssim 1$, it is the best value that we can get as observed scatter is slightly less than that predicted by the analytical uncertainties.

Tab. S1. Parameters from the fitting results of the XRD peaks in Figure S1.

	Value	Standard	Error	t-Value	Prob > |t|	Dependency
Baseline	y_0	-0.15	0.01	-13.91	0	0.54
Peak 1	x_c	5.52	1.29×10^{-4}	42861.48	0	0.21
	A	8.33	0.05	172.43	0	0.84
	w	0.08	3.23×10^{-4}	241.83	0	0.57
	μ	0.07	0.02	3.86	1.16×10^{-4}	0.88
Peak 2	x_c	10.76	0.06	215.12	0	0.05
	A	-23.35	0.63	-36.82	0	0.67
	w	5.04	0.09	53.81	0	0.45
	μ	2.35	0.02	115.94	0	0.19
Peak 3	x_c	16.99	4.80×10^{-4}	3542.84	0	0.61
	A	0.62	0.05	11.67	0	0.79
	w	0.09	7.50×10^{-4}	11.59	0	0.66
	μ	0.57	0.26	2.25	1.02×10^{-4}	0.88
Peak 4	x_c	18.54	3.41×10^{-3}	5432.07	0	0.34
	A	1.53	0.05	33.44	0	0.41
	w	0.06	2.47×10^{-3}	23.74	0	0.58
	μ	1.98	0.04	50.27	0	0.43
Peak 5	x_c	24.85	1.79×10^{-3}	13886.78	0	0.44
	A	2.26	0.04	60.28	0	0.43
	w	0.04	8.24×10^{-4}	45.05	0	0.56
	μ	2.01	0.02	96.79	0	0.40
Peak 6	x_c	31.05	2.88×10^{-3}	10800.59	0	0.65
	A	2.27	0.06	37.52	0	0.75
	w	0.13	3.72×10^{-3}	34.41	0	0.65
	μ	0.63	0.09	7.14	1.05×10^{-4}	0.88
Peak 7	x_c	37.35	3.04×10^{-3}	12295.11	0	0.67
	A	2.29	0.07	34.53	0	0.82
	w	0.15	3.21×10^{-3}	46.01	0	0.55
	μ	0.22	0.10	2.08	1.84×10^{-4}	0.91

Tab. S2. Calculated lattice and geometrical parameters of $(CH_3(CH_2)_7NH_3)_2SnBr_4$[1], $(C_6H_5(CH_2)_2NH_3)_2SnBr_4$[2], and $(C_6H_5CH_2NH_3)_2SnBr_4$ (this work).

Compounds	*Structure*	a(Å)	b(Å)	c(Å)	α(°)	β(°)	γ(°)	*Ref.*
$(CH_3(CH_2)_7NH_3)_2SnBr_4$	Orthorombic	8.436	8.166	38.381	90	90	90	this work
$(CH_3(CH_2)_7NH_3)_2SnBr_4$	Orthorombic	8.982	8.688	37.482	90	90	90	1
$(C_6H_5(CH_2)_2NH_3)_2SnBr_4$	Triclinic	11.538	11.601	17.511	80.4	74.5	89.9	this work
$(C_6H_5(CH_2)_2NH_3)_2SnBr_4$	Triclinic	11.558	11.623	17.313	80.3	74.3	89.8	2
**$(C_6H_5CH_2NH_3)_2SnBr_4$**	**Triclinic**	**11.512**	**11.321**	**16.850**	**80.4**	**74.7**	**90.5**	**this work**

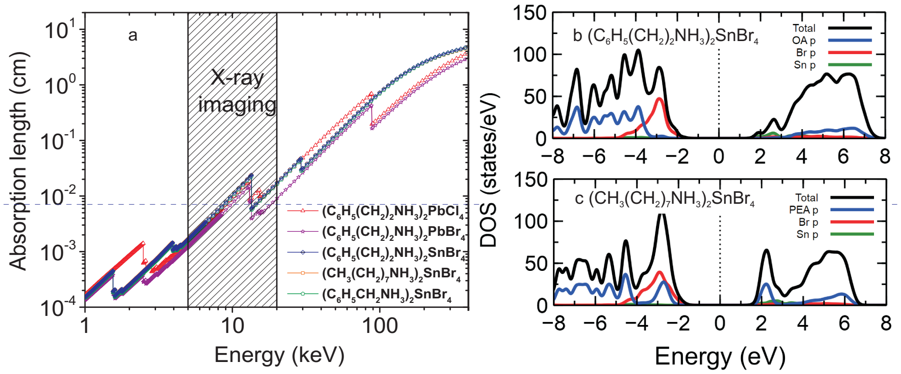

Fig. S2. a) Calculated absorption lengths as a function of photon energy, covering the X-ray spectral region for $(C_6H_5(CH_2)_2NH_3)_2PbCl_4$, $(C_6H_5(CH_2)_2NH_3)_2PbBr_4$, $(C_6H_5(CH_2)_2NH_3)_2SnBr_4$, $(CH_3(CH_2)_7NH_3)_2SnBr_4$, and $(C_6H_5CH_2NH_3)_2SnBr_4$. b) Total (black) and projected (color) density of states (DOS) of $(C_6H_5(CH_2)_2NH_3)_2SnBr_4$ and $(CH_3(CH_2)_7NH_3)_2SnBr_4$. Blue, green, and red lines represent $C_6H_5(CH_2)_2NH_3)_2$ (or $CH_3(CH_2)_7NH_3)_2$) s and p, Sn p, and Br p orbitals, respectively.

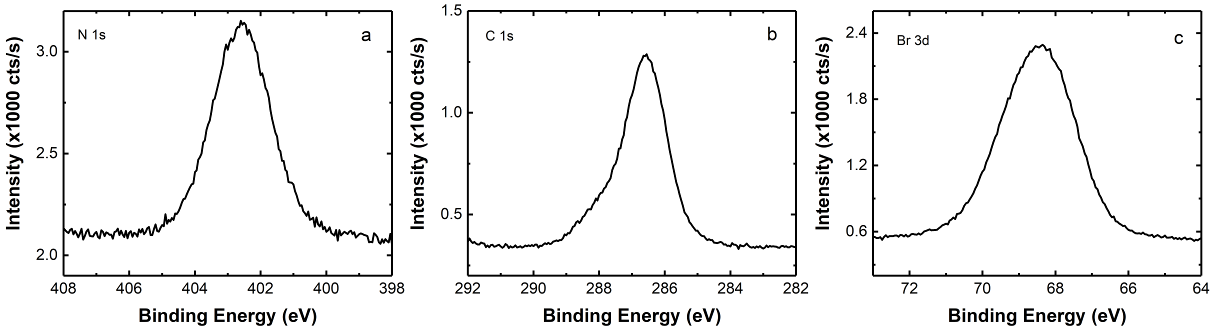

Fig. S3. XPS spectra of a) N 1s, b) C 1s and c) Br 3d of $(C_6H_5CH_2NH_3)_2SnBr_4$.

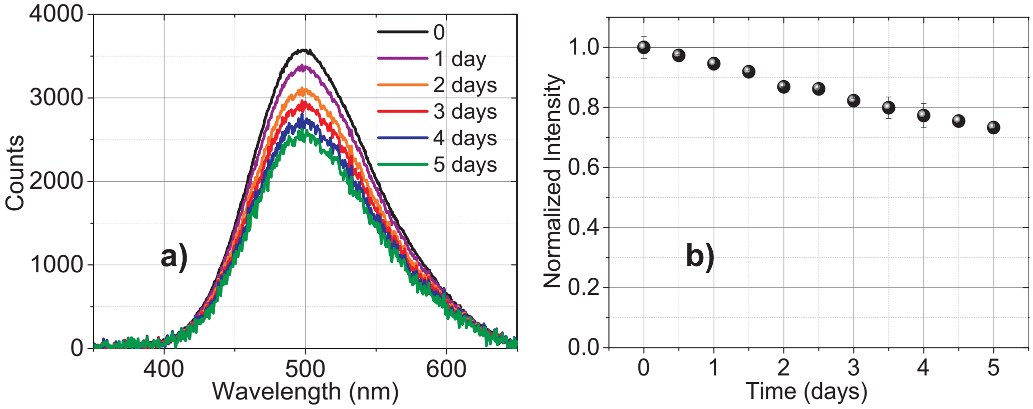

Fig. S4. a) Photoluminescence (PL) spectra and b) PL intensity as function of air exposure day of $(C_6H_5CH_2NH_3)_2SnBr_4$ for 5 days. The excitation is at 266 nm with 30 kHz picosecond pulsed diode laser.

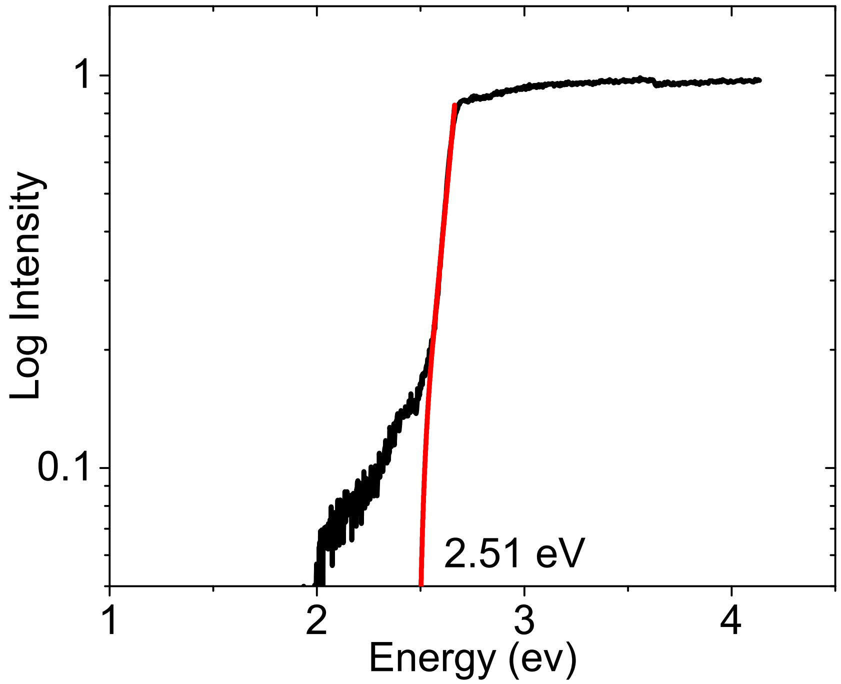

Fig. S5. The optical bandgap determined through the Tauc plot of absorption spectra of $(C_6H_5CH_2NH_3)_2SnBr_4$.

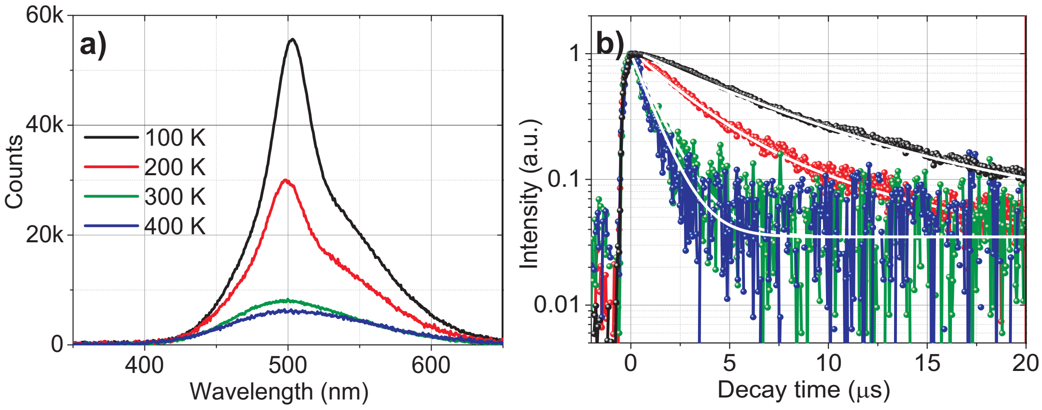

Fig. S6. Temperature dependent a) PL spectra and b) Time-Resolved PL (TRPL) decay curves of $(C_6H_5CH_2NH_3)_2SnBr_4$ recorded from 100 to 400 K with an interval 100 K. The excitation wavelength is 266 nm while for the decay curves, the monitoring wavelength is 500 nm. The white lines in b) are the exponential fits of the curves. The lifetimes at 100, 200, 300, and 400 K are 8.75, 4.59, 1.05, and 1.01 μs, respectively.

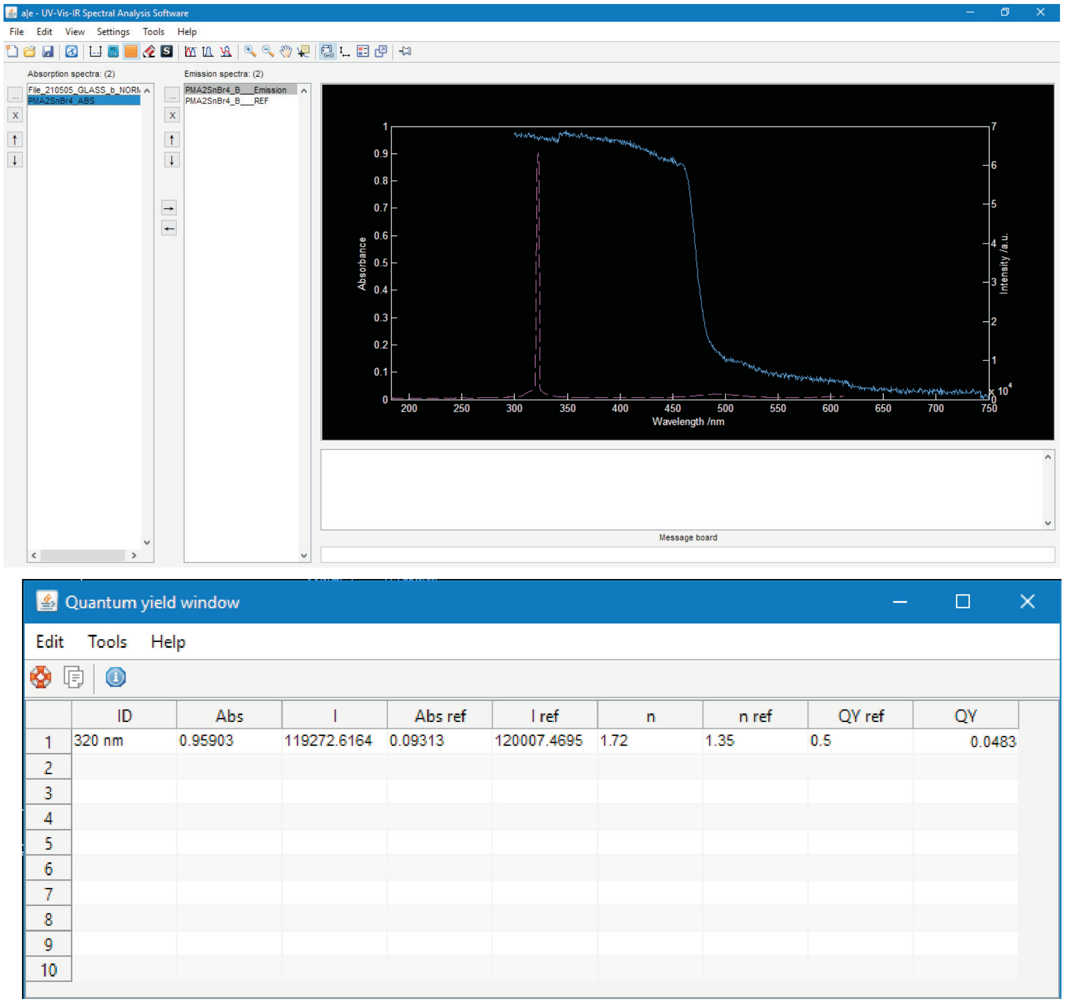

Fig. S7. Screenshot of Fluorotools freeware to calculate PL quantum yield (PLQY) of $(C_6H_5CH_2NH_3)_2SnBr_4$ based the inputs from the glass reference absorption, the absorption, and the PLQY spectra of the crystals.

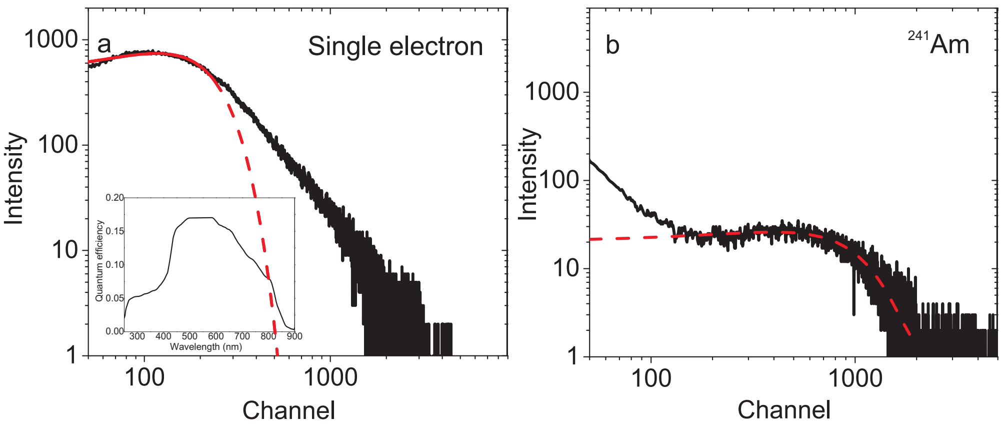

Fig. S8. a) Measured single electron response of the Hamamatsu R9880U-20 photomultiplier tube (PMT). The inset shows the quantum efficiency spectra. b) Pulse height spectra under gamma-ray excitation of ^{241}Am source at 59.5 keV. The photoelectron yield from comparing the photo peak and the single electron response peak with their gain is 585 photoelectron/MeV. Using quantum efficiency of 0.16, the light yield is 3600 photons/MeV. The energy resolution is 79.40%.

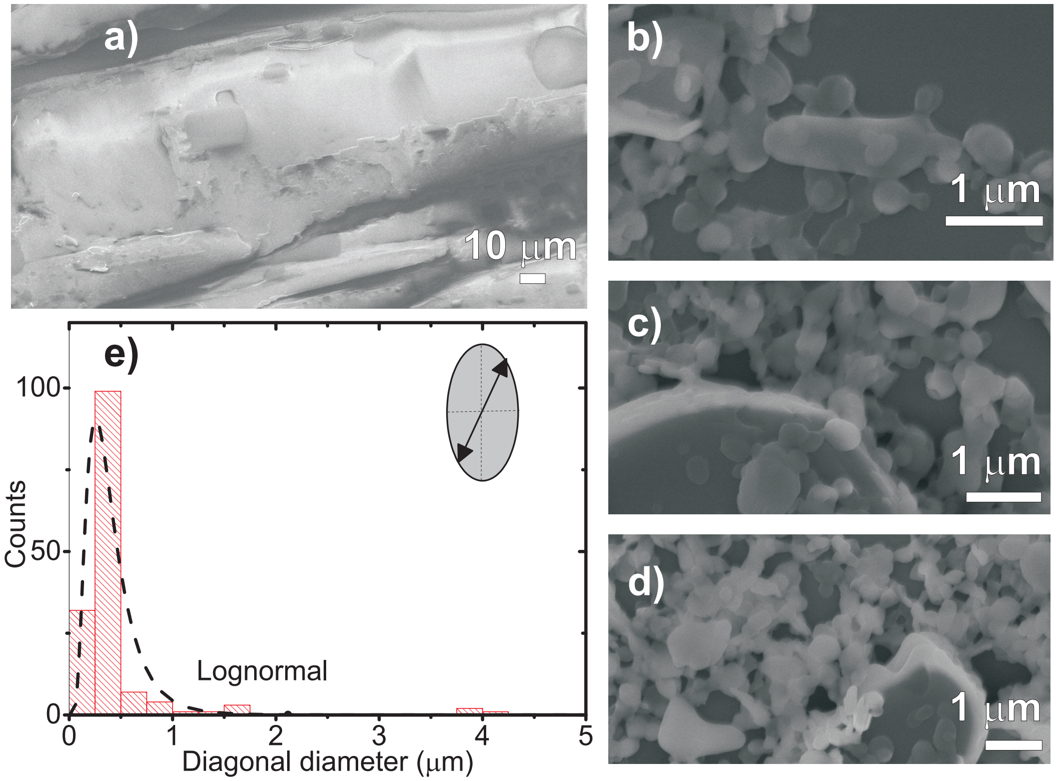

Fig. S9. $(C_6H_5CH_2NH_3)_2SnBr_4$ microcrystals and their size distribution. a) Scanning electron microscope (SEM) image of the surface of single crystals showing relatively smooth surfaces of the crystals. b, c, d) SEM images of the microcrystals after the ultrasonic bath. e) Sizes of the microcrystals with their lognormal distribution.

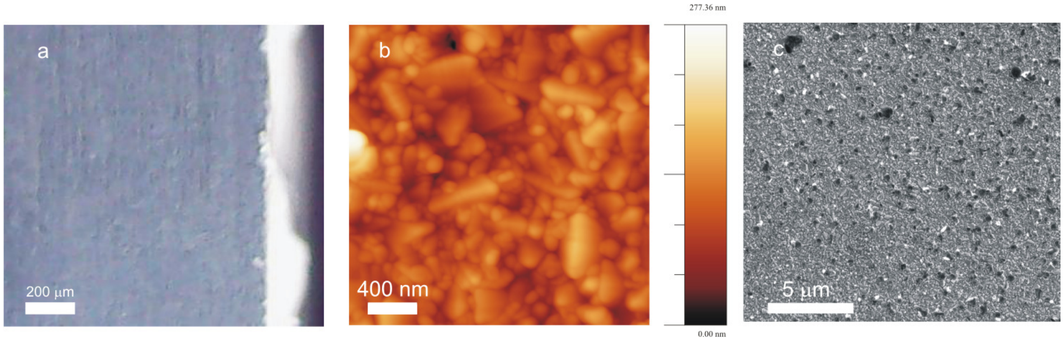

Fig. S10. a) Cross-section microscope, b) tapping mode atomic force microscope (AFM), and c) SEM images for $(C_6H_5CH_2NH_3)_2SnBr_4$ microcrystal film with a thickness of 155 ± 23 μm. The film is prepared from the solution of microcrystals and 2 ml Poly(methyl methacrylate) (PMMA 5% concentration). The solution was spin-coated on a UV-ozone treated cover glass substrate with 500 rpm for 60 s (acceleration: 100 rpm per s).

Other data can be obtained through corresponding authors.

REFERENCES

[1]J. Cao, Z. Guo, S. Zhu, Y. Fu, H. Zhang, Q. Wang, and Z. Gu, ACS Appl. Mater. Interfaces **12**, 19797 (2020).

[2]L.-J. Xu, H. Lin, S. Lee, C. Zhou, M. Worku, M. Chaaban, Q. He, A. Plaviak, X. Lin, B. Chen, M.-H. Du, and B. Ma, Chem. Mater. **32**, 4692 (2020).
